# ‘My first thoughts are…’: a Framework Method analysis of UK general practice healthcare professionals’ internal dialogue and clinical reasoning processes when seeing patients living with obesity in primary care

**DOI:** 10.1136/bmjopen-2024-086722

**Published:** 2025-04-02

**Authors:** Sarah Serjeant, Sally Abbott, Helen Parretti, Sheila Greenfield

**Affiliations:** 1 Coventry University, Coventry, UK; 2 Department of Dietetics, University Hospitals Coventry and Warwickshire NHS Trust, Coventry, UK; 3 Norwich Medical School, University of East Anglia, Norwich, UK; 4 Institute of Applied Health Research, University of Birmingham, Birmingham, England, UK

**Keywords:** Primary Health Care, Obesity, QUALITATIVE RESEARCH, Clinical Reasoning

## Abstract

**Objectives:**

To use vignettes to facilitate exploration of the internal dialogue and clinical reasoning processes of general practice healthcare professionals (GPHCPs) during interactions with patients living with obesity.

**Design:**

This study used an exploratory qualitative research design. Data were collected using semistructured interviews. Interviews were transcribed verbatim, and data analysed using Framework Method analysis. Five vignettes were presented to participants, showing a patient’s photograph, name, age and body mass index. Participants were asked to describe their first impressions of each fictionalised patient.

**Setting:**

Interviews were conducted remotely via Skype between August and September 2019.

**Participants:**

A convenience sample of UK GPHCPs was recruited via a targeted social media strategy, using virtual snowball sampling. 20 participants were interviewed (11 general practice nurses and 9 general practitioners).

**Results:**

Five themes were generated: visual assessment, assumed internal contributing factors, assumed external contributing factors, potential clinical contributing factors and potential clinical consequences. A pattern-recognition approach was identified, as GPHCPs’ assumptions around patients’ lifestyles, occupations and eating habits emerged as explanations for their weight, with a mixture of both objective and subjective comments.

**Conclusions:**

While it is part of the diagnostic skill of a clinician to be able to form a clinical picture based on the information available, it is important to be aware of the potential for assumptions made within this process to contribute to unconscious bias/stereotyping. Healthcare professionals need to work to counteract the potential impact of internal bias on their consultations to provide fair and equitable care for people living with obesity, by exercising reflexivity within their clinical practice.

**STRENGTHS AND LIMITATIONS OF THIS STUDY**

- Conducting remote interviews allowed data to be collected from a diverse demographic of participants, although this could have hindered the detection of non-verbal cues, potentially losing some of the nuance of the discussion.
- The use of patient vignettes facilitated the exploration of a sensitive topic, although fictionalised information may constrain the transferability of the findings to real-world consultations.
- The systematic procedure of the Framework Method and embedding peer review enhances dependability.
- Data collection was conducted in 2019, and perspectives may have evolved since then, potentially limiting the transferability of the findings to the present day.

## Introduction

 Obesity is classified as having a body mass index (BMI) ≥30 kg/m^2^.^1^ In 2021–2022, 25.9% of adults aged ≥18 years in England were estimated to be living with obesity.^2^

In the UK, general practice is often the access point for the diagnosis and management of chronic diseases, including obesity and related comorbidities. When a person is living with obesity, National Institute for Health and Care Excellence^1^ guidance suggests healthcare professionals (HCPs) should make an initial assessment, taking anthropometric measurements and discussing the implications of the person’s weight; and use their clinical judgement to investigate comorbidities and other factors, considering the person, the timing of the assessment, the degree of obesity and results of previous assessments.

Compared with those with a healthy weight, people living with obesity are at higher risk of developing multiple chronic conditions.^3^ Multimorbidity is considered a major challenge in primary care, placing pressure on general practice healthcare professionals (GPHCPs).^4^ However, it is important that HCPs avoid assuming all symptoms reported by patients living with obesity are weight-related, as patient perspectives reveal this leaves them feeling dismissed.^5^

A recent systematic review,^6^ exploring general practitioners’ (GPs) clinical reasoning when managing patients with multimorbidity, found that they often struggled to explain the clinical reasoning used, despite this being central to practice. GPs described their reasoning as intuitive, however, results highlighted an analytical process of balancing risks and benefits for the patient.

Attempts to understand clinical reasoning have led to the development of several models. Research by Yazdani *et al*^7^ critically reviewed six existing clinical reasoning models in the context of general practice: the hypothetico-deductive model,^8^ pattern recognition,^9^ a dual process diagnostic reasoning model,^10^ pathway for clinical reasoning,^11^ an integrative model of clinical reasoning^12^ and the model of diagnostic reasoning strategies in primary care.^13^ The authors concluded that while these models each shed light on different elements of the clinical reasoning process, models specific to general practice are still required to assist GPs with the specific features of primary care and challenges for clinical reasoning in this setting.

The thought processes underpinning clinical reasoning are often internal and extremely fast, making these difficult for both clinicians and researchers to access and understand.^6^ Research specific to primary care and the unique challenges for clinical reasoning in this setting is limited. Hence, the aim of this study was to use vignettes to explore the values, beliefs and norms that underlie the internal dialogue and clinical reasoning processes of GPHCPs when seeing patients living with obesity.

## Methods

This study used an exploratory qualitative research design.^14^ Data for this study were collected as part of one interview study with two aims. First, to explore GPHCPs’ experiences of referring patients with obesity to dietitians and their perceptions of the value and practicalities of embedding dietitians within the general practice team, as reported by Abbott *et al.*^15^ Second, to use vignettes to facilitate exploration of the internal dialogue and clinical reasoning processes of GPHCPs when seeing patients living with obesity. This paper focuses on the reporting of the secondary aim.

### Sampling strategy

General practice nurses (GPNs) and GPs practising in the UK were eligible to take part in the interview study.^15^ A convenience sample of GPHCPs was recruited using online social networks via virtual snowball sampling, whereby a small pool of social media followers nominated other participants who met the eligibility criteria. Recruitment took place between August and September 2019, via online advertisement on the platforms of Facebook, Twitter (now X) and LinkedIn. Readers of the advertisement were encouraged to forward it to eligible participants within their networks, to support virtual snowball sampling. After reading the online participant information sheet, participants confirmed their written consent electronically, provided demographic screening information, and were contacted to arrange an interview time. None of the researchers had any prior relationship with participants.

### Data collection

Semistructured interviews were carried out by one researcher (SA), using a topic guide (see online supplemental information 1) and five vignettes which were presented to participants. These vignettes were fictionalised patients with different genders, ethnicities and a range of BMIs >30 kg/m^2^. Participants were shown a patient’s photograph, name, age and BMI for each vignette, and they were asked to imagine these patients had come for a consultation to discuss a medical concern which was not necessarily obesity and to describe their first impressions of each patient.

Participants were given the choice for the interview to be conducted by Voice over Internet Protocol (VoIP, ie, voice communication over the internet instead of via phone lines)^16^ using Skype, or face-to-face. Interviews were audio-recorded, anonymised and transcribed verbatim by a professional transcription service. Each transcript was checked for accuracy by the interviewer (SA) prior to analysis.

### Data analysis

The Framework Method^17^ was used for data analysis; a systematic method of categorising and organising qualitative data, commonly used to analyse semistructured interview transcripts.^17^ This method is not aligned to a particular paradigm but allows flexibility for use with many qualitative approaches which aim to generate themes.^17^ It follows a structured seven-stage process: transcription, familiarisation, coding, developing an analytical framework, applying the framework, charting data into the framework matrix and interpreting the data. One researcher (SS) independently read all the transcripts to become familiar with the interviews. Three transcripts were then read line-by-line, and codes were applied to identify interpretations. After coding these initial transcripts, the research team met to develop a working analytical framework using emergent data. This framework was then applied by one researcher (SS) to the remaining transcripts and a spreadsheet was used to chart the data into a matrix. The research team then reviewed the matrix and discussed the interpretation of the data.

### Reflexivity

The researchers’ own experiences of obesity management and their professional identities (SS and SA as dietitians; HP as a GP and SG as a medical sociologist) have been considered within the research process. Regular peer discussion was essential to help examine existing understanding and assumptions, identify potential biases and ensure the themes were representative of the participants’ perspectives.

### Patient and public involvement

Patients and/or the public were not involved in the design or conduct or reporting or dissemination plans of this research.

## Results

24 GPHCPs consented to participate in the interviews:^15^ two withdrew their consent due to unavailability and a further two participants were uncontactable. Therefore, in total, 20 GPHCPs (11 GPNs and 9 GPs) were interviewed. All elected to be interviewed using VoIP. Interviews lasted an average of 41 min (range 24–61 min). The data were considered to have reached saturation with 20 participants because no new insights were revealed. Most participants were female (18/20) and held a variety of positions, with general practice experience ranging from 3 to 30 years. Participants worked across small, large, urban and rural general practices with diverse patient demographics across England and Scotland (Index of Multiple Deprivation scores for their practices ranged from 1 to 9;^18^ non-white ethnicities in their patient populations ranged from 1.5% to 61.1%) (see table 1).

**Table 1:** Participants’ demographics and employing general practices’ patient population demographics

Participant characteristics	n=20	Median (IQR)
Profession		
GP	9	N/A
GPN	11	
Gender		
Male	2	N/A
Female	18	
Experience (years)		
1–5	3	15.5 (10.0)
6–10	2	
11–15	5	
16–20	6	
21–25	1	
26+	3	
Position		
Salaried	2	N/A
Locum	2	
Partner	5	
GPN manager	4	
GPN	3	
ANP	3	
GPN educator	1	
**Employinggeneralpractice**		
Size of practice*		
Small	6	N/A
Large	12	
Locum so >1 GP practice	1	
Not available†	1	
Location of employing general practice		
Rural	4	N/A
Urban	15	
Locum so >1 GP practice	1	
Deprivation decile^18^		
1 (most deprived)	1	5 (4)
2	3	
3	2	
4	2	
5	3	
6	2	
7	2	
8	0	
9	2	
10 (least deprived)	0	
Locum so >1 GP practice	1	
Not available†	1	
Not available‡	1	
Non-white ethnicity (%) of patient population		
1–5	9	5.5 (12.9)
6–10	3	
11–15	1	
16–20	1	
21–25	2	
>25	1	
Locum so >1 GP practice	1	
Not available†	1	
Not available‡	1	

* Small practices <6000 registered patients and large practices ≥6000 registered patients.

† Data not available for non-National Health Service GP practices.

‡ Data not available for Scotland.

ANPadvanced nurse practitionerGPgeneral practitionerGPNgeneral practice nurse

**Figure 1:**
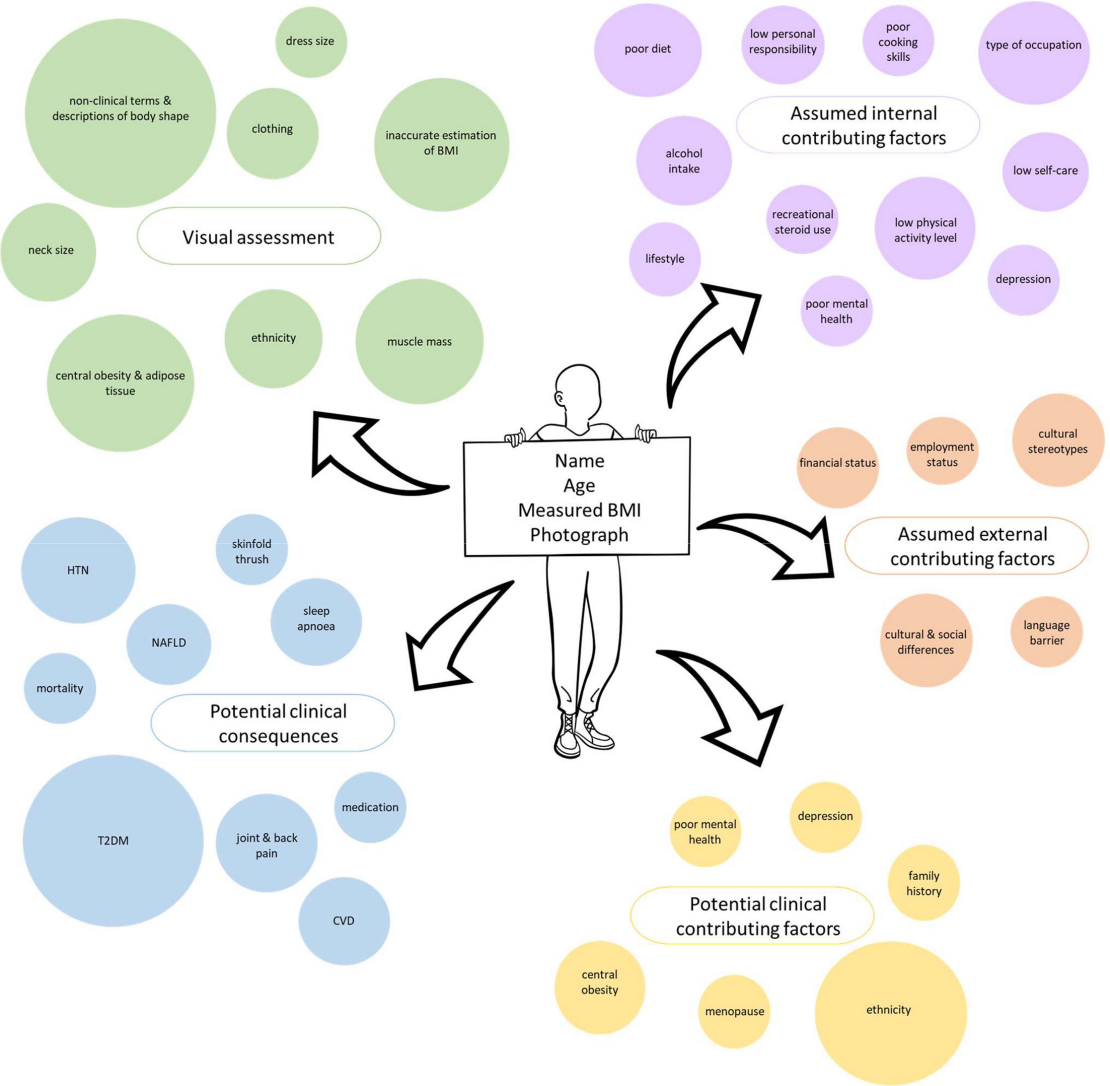
Concept Map. BMI, body mass index; CVD, cardiovascular disease; HTN, hypertension; NAFLD, non-alcoholic fatty liver disease; T2DM, type 2 diabetes mellitus.

Data were organised into five main themes: visual assessment, assumed internal contributing factors, assumed external contributing factors, potential clinical contributing factors and potential clinical consequences, as seen in figure 1. The findings within these themes are described below.

### Visual assessment

GPHCPs first used the information provided to form visual assessments of the patients. This included both objective and subjective comments on their appearances. The term ‘central obesity’ was used regularly to describe the distribution of adipose tissue noted from the photographs. Neck size and muscle mass were also taken into consideration as part of the visual assessment, and conclusions were drawn about ethnicity based on the names and photographs provided.

Well it looks like he’s from an ethnic minority group and Khan would suggest that. He looks like he’s got some central obesity… GP3, discussing Mr Khan

There were also subjective assessments made based on appearances; for example, using non-clinical descriptors of body shape. Language such as ‘big chap’, ‘cuddly’, ‘pudgy’ and ‘chunky’ was used to describe the patients.

I mean…she’s got a neat waist, she’s a bit chunky in the legs… GPN11, discussing Ms Banda

Clothing choices were influential on GPHCPs’ assessments, especially for female patients. It was implied that as the female patients were well presented, they must ‘take pride’ in their appearance. GPHCPs also felt clothing choices made it difficult to visually assess weight and BMI, and some remarked that they would have likely underestimated BMI based on visual assessment alone. Consequentially, GPHCPs tended to decide that they would not be likely to discuss obesity with the female patient vignettes.

Clearly, image…is very important. They’re dressed very well. You’re not going to be concerned that they’re not looking after themselves in that sense. GPN8, discussing Ms Banda & Ms Jarvis

Yeah, she is a bit overweight. I’m not discussing anything with her because she’s got a very curvy figure and she’s not hiding it. But she could do with losing a bit of weight. GP7, discussing Ms Banda

Just because I think it’s really difficult to…I think with what they’re wearing you wouldn’t necessarily…unless you weighed them, I wouldn’t know that their BMIs were…what they’re wearing is important. GP9, discussing Ms Banda & Ms Jarvis

### Assumed internal contributing factors

Using the information provided, GPHCPs tried to build a picture of each patient, using assumptions about their backgrounds, history and various factors that could have contributed to their obesity. GPHCPs made assumptions about the patients’ attitudes and understanding of their obesity along with their lifestyles, citing low physical activity levels, poor diet and cooking skills as likely contributors.

And I suspect that he doesn’t do his own cooking. GP2, discussing Mr Khan

He looks like he is quite, probably quite sedentary, his lifestyle, yeah. GPN4, discussing Mr Madgwick

Poor mental health, lack of personal responsibility and low levels of self-care were also cited as likely contributing factors.

I would expect that if I were talking to him about his weight he would say something like ‘I've always been big boned’. That drives me a bit bonkers really when they say big boned. GP2, discussing Mr Madgwick

### Assumed external contributing factors

External contributing factors were also considered by GPHCPs, and assumptions were formed about hobbies, occupation and financial status. GPHCPs created backstories for the patients to help explain their situations.

…Is it because he’s a builder, and every Friday night they all go to the pub after work and have ten pints, or they all go out for a curry, and have a lot of carbohydrates for lunch, and fried breakfasts? GPN9, discussing Mr Madgwick

Oh, he’s a big man…so he might be a bodybuilder, he might work out at the gym. GPN4, discussing Mr Robinson

He doesn’t have a job. He’s not working and is he poor? So is he eating all the wrong sorts of foods? Or he’s super-rich and he’s just sat at a desk earning lots of money. GP8, discussing Mr Madgwick

Cultural aspects were also considered by GPHCPs, including the need to consider the impact of cultural differences and potential language barriers as part of the assessment. Assumptions about the cultural background of the patient vignettes and their presumed dietary preferences and attitudes towards obesity were also made.

She looks like, and based on her name, I imagine she’s probably a Roma lady. So I would be wanting to discuss possibilities of the cultural aspect of being Roma, and all the social things that go with that. GPN7, discussing Ms Banda

My first thoughts are, he thinks that that’s okay, because culturally, if they’re overweight, it’s seen that they’re richer, and that’s really important within their culture and community – to be seen as successful. GPN9, discussing Mr Khan

His, probably, carb ratio is so high. His carbs and proteins metabolic ratios are so high within his diet, then that’s all just racial profiling to be fair. GP8, discussing Mr Khan

When I've talked to Afro-Caribbean people about food and their eating it tends to be high salt, high fat, and high sugar. That’s difficult to unravel. GP2, discussing Mr Robinson

Confidence regarding understanding and managing cultural differences impacted the GPHCPs’ assessment plans and the likelihood of discussing weight.

I suppose the other thing, I don't have a large BME community…and the BME community I've got are all professionals, so it is a bit an atypical population maybe. …My exposure to BME communities in terms of patients is limited. So understanding their cultural issues, you know is very much second-hand or third hand, is my experience. How well I am going to navigate that? GP1, discussing Mr Khan

But the good thing is with him, because I’m Asian I’d be able to easily tackle that and say, Look what are you eating? Are you eating rice, naan and whatever for afters? I’d be like, ‘Well you need to cut those down.’ …I’m probably more confident in tackling obese Asians definitely than white obese people or black obese people… Just because of my race. GP8, discussing Mr Khan

### Potential clinical contributing factors

Clinical contributing factors were also discussed by participants. Ethnicity and family history were often highlighted as non-modifiable risk factors which would impact assessment and risk of obesity.

You know again he’s got central obesity, you know because of his ethnic origin you're thinking of comorbidities… GP1, discussing Mr Robinson

…Middle aged and he’s got a high BMI and…he’s of Asian origin so you’d immediately sort of think that he’s at higher risk of things like diabetes… GP5, discussing Mr Khan

One of the questions with any Asian patients is ‘who in your family have got diabetes’ and they’ll reel off like, 25 people in their family, so that’s easy enough for me to tackle. GP8, discussing Mr Khan

The distribution of weight identified by GPHCPs’ visual assessment was also raised as a clinical contributing factor for concern.

I would look at abdominal obesity for him really. I think he’s got quite a large waist circumference, so therefore probably quite a high risk…for diabetes. GPN3, discussing Mr Khan

Mental health concerns were only raised by one GPHCP.

We’d need to know what else is going on because a 29-year-old, why has he got that sort of BMI? Has he got anything else that’s going on that we need to know about? …Is he depressed? GPN2, discussing Mr Madgwick

The impact of menopause was also considered by some as a contributing factor.

If she’s perimenopausal would be the only other thing that I’d add for this lady, which can obviously affect her weight as well. GPN5, discussing Ms Jarvis

### Potential clinical consequences

GPHCPs expressed concern around the clinical consequences of obesity for each patient vignette. Type 2 diabetes mellitus was the main concern, along with hypertension, joint and back pain, and non-alcoholic fatty liver disease.

Well I'd expect him to be diabetic and having difficulty with his sugar control. I would expect knee, ankle, hip, back problems. I'd also expect to find fatty liver. GP2, discussing Mr Robinson

The risk of cardiovascular disease was also a concern expressed by the GPHCPs.

…I am thinking he’s a heart attack waiting to happen…. GP1, discussing Mr Khan

The visual assessment of neck size was linked to the risk of sleep apnoea.

Very, very high BMI and I would have thought he’d be a prime candidate for sleep apnoea looking at the size of his neck. That’s my first impression. GP6, discussing Mr Robinson

GPHCPs felt confident in being able to address these clinical concerns and cited they would use these to open the discussion around weight, rather than addressing weight directly.

…If they are developing problems there’s a way in, I suppose, for me to then maybe talk about their weight because it’s easier for me to talk from the medical perspective and you know in a less confrontational way…GP1, discussing Mr Madgwick

I definitely wouldn’t tackle a female head-on just because I’d be so worried about…insulting women with their weight… GP8, discussing Ms Banda & Ms Jarvis

## Discussion

This qualitative study used vignettes to facilitate exploration of the internal dialogue and clinical reasoning processes of GPHCPs when seeing patients living with obesity. Five themes were generated: visual assessment, assumed internal contributing factors, assumed external contributing factors, potential clinical contributing factors and potential clinical consequences. GPHCPs used the information provided to build a backstory for each patient, to try and explain the possible causes and consequences of their weight. Factors such as ethnicity, lifestyle, socioeconomic status and family medical history were all considered, with a mixture of both objective and subjective comments.

### Comparison with previous literature

The themes reflect the internal dialogue described by GPHCPs. GPHCPs were presented only with a photograph, name, age and measured BMI within the vignettes, and from these emerged assumptions around these patients’ lifestyles, occupations and eating habits. This suggests a pattern-recognition model of clinical reasoning, as GPHCPs recognised key features in the vignettes and matched these with a pattern previously formed in their memories.^7^ This is a rapid, non-analytical process often used when patients present with problems encountered by clinicians on a regular basis.^7^

However, it is hard to say whether this pattern-recognition process stems from patterns of clinical signs and symptoms, unconscious biases/stereotypes or both. It is recognised that obesity is a multifactorial disease with complex genetic, behavioural, socioeconomic and environmental origins.^19^ However, a cross-sectional mixed methods study^20^ found that both patients and primary care practitioners scored behavioural factors as significantly more important for causing obesity than medical, psychological or social factors. This perception is also reflected in a qualitative systematic review,^5^ which found that doctors often assumed a person who was overweight must have an unhealthy diet. These findings reflect a misconception that the main causes of obesity are all within an individual’s control, an assumption which could fuel negative stereotypes/attitudes and ultimately drives weight stigma.

While similar assumptions around diet and physical activity are reflected within the ‘assumed internal contributing factors’ theme, GPHCPs in this study also showed good awareness and understanding of the non-modifiable risk factors and comorbidities related to obesity. This suggests a mixture of both clinical experience and stereotyping within the pattern-recognition process.

Another finding was a lack of confidence in visually assessing weight and BMI. GPHCPs were provided with a measured BMI in each vignette, and many acknowledged that they would have underestimated BMI. Similarly, an experimental study with trainee and qualified GPs^21^ found a tendency to underestimate BMI based on visual assessment of photographs, and this was more pronounced the higher the photographed subject’s actual BMI. This underestimation was associated with a lower intention of discussing weight management. While these findings are based on photographs rather than in-person visual assessment, which could reduce the transferability to a face-to-face environment, it still highlights the importance of taking anthropometric measurements rather than relying on visual estimations.

GPHCPs in the present study were open about their concerns regarding discussing weight with patients, particularly those of a different gender or culture to their own, and felt the best approach was to use clinical concerns to open the discussion. This was also identified by McHale *et al*,^20^ as primary care practitioners expressed a clear preference for discussing weight within the context of patients’ existing health issues that could be directly related to weight, as they believed they had supporting medical evidence to justify the relevance of discussing weight. Broaching weight without a clear health-related reason was deemed inappropriate, risking a negative emotional reaction from patients. A similar approach was revealed in a qualitative systematic review,^22^ where GPHCPs described linking discussions of weight to relevant medical concerns, seeing the topic easier to broach with patients when positioned as more ‘doctorable’.

While discussing weight without clear evidence of an associated medical comorbidity was feared as risking a negative response from the patient, evidence suggests that clinical inertia regarding overweight and obesity can also be detrimental. Findings from Ananthakumar *et al*^5^ showed that patients viewed omission of weight-related discussion as a sign of judgement, as some perceived the doctor’s silence meant they were ‘unworthy of medical time’. A survey of 1500 people in the UK living with obesity found that, of those who previously had a weight-related conversation with their HCP, 65% were happy this was raised. Of those who had not previously had a weight conversation, 58% would have liked this to have been raised.^23^ This suggests patients may be more open to weight-related discussions than HCPs perceive.

It is essential that weight-related discussions are handled in a sensitive manner, free from bias and stigma, to avoid leaving patients feeling embarrassed, blamed, discouraged or offended.^23^ Some phrases used by GPHCPs in this study indicate the presence of weight bias/stigma. While these comments may not reflect the language used when speaking directly to patients, this internal dialogue could influence GPHCP decision-making.^24^ Existing literature suggests that the explicit and implicit negative attitudes held by primary care providers (and other HCPs) about people with obesity influence communication (both verbal and non-verbal) and decision-making within clinical interviews, resulting in a less patient-centred approach.^25^ While it is part of the diagnostic skill of a clinician to be able to form a clinical picture based on the information available to them, it is important to be aware of the potential for assumptions to lead to unconscious bias/stereotyping within this process, and exploration of the true clinical implications of these attitudes should be explored further within future research.

### Strengths and limitations

The Consolidated Criteria for Reporting Qualitative Research checklist^26^ was used to aid transparent reporting of the research process (see online supplemental information 2). The systematic procedure of the Framework Method is a recognised methodology, which further enhances dependability by aiding transparent reporting of the steps taken during data analysis and embedding peer review.^17^ The matrix structure used as part of the Framework Method also helped to improve credibility by facilitating the identification of patterns in the data. The use of open coding created a more inductive approach, allowing themes to be generated from the emerging data and facilitating full exploration of the research topic. It is acknowledged that the characteristics of the researchers may have influenced data analysis, however, reflexivity has been transparently reported to improve confirmability.

Using VoIP allowed data to be collected from a diverse demographic of participants and from multiple geographical areas across the UK, increasing transferability of the findings; however, the experiences of GPHCPs in other countries and healthcare systems outside of the National Health Service may differ. Participants were able to choose whether to make themselves visible via video during interviews; however, with or without the use of video, VoIP can hinder the detection of non-verbal cues compared with face-to-face interactions, and potentially lose some nuance of the discussion.

This study adds evidence to the limited body of evidence currently available on clinical reasoning within primary care settings. However, the thought processes underpinning clinical judgement and reasoning are complex and difficult to access and understand for both clinicians themselves and researchers,^6^ therefore, it is unlikely that the full intricacies of the clinical reasoning process have been captured within this one study. While the vignettes provided a less personal way to explore a potentially sensitive topic, the use of photographs and fictionalised information could reduce the transferability of the findings to face-to-face consultations. It is also worth noting that data collection took place in 2019, therefore, opinions on this topic could have changed since then. Future research should explore whether stereotyping of patients with obesity influences the quality of clinical care provided by GPHCPs.

It is also important to consider the influence of the researchers on the participants. The interviewer (SA) is also a HCP and therefore could have been considered a peer by participants. It may also be pertinent to note that SA is not living with overweight or obesity, which would have likely been visible to the participants during the Skype interviews. If participants considered the interviewer to share characteristics with the vignettes, this could have hindered candid discussion; participants may not have felt comfortable verbalising some of the more subjective comments from their internal dialogue, due to fear of causing offence. Therefore, the characteristics of the interviewer could have contributed to participants’ open discussions and potentially increased the credibility of the findings.

### Implications and application

The findings suggest that GPHCPs make assumptions about patient characteristics based on visual appearance, some of which are objective, however, other aspects could stem from unconscious bias/stereotyping.

It is accepted and acknowledged that the disciplinary training, scholarly knowledge, values and sociocultural context of researchers shape the qualitative research process, and arguably the same applies to the clinical reasoning process. Everyone has internal biases; however, within healthcare settings, this may influence subsequent clinical reasoning and management decisions. Therefore, to provide fair and equitable care, HCPs need to have a heightened awareness of their biases and work to counteract their potential impact on consultations.

Professional bodies recognise the need for regular reflective practice.^27 28^ Reflexivity, while related, is a more dynamic process. Reflection is a deep review of events, whereas reflexivity requires ‘finding strategies to question our own attitudes, theories in use, values, assumptions, prejudices and habitual actions; to understand our complex roles in relation to others’.^29^ It is a continuous process of observing and questioning personal biases and social/cultural norms. While this is usually associated with qualitative research, it could also be harnessed as an educational approach for HCPs, integrated into medical training and continued professional development. Using this as a process to help reveal unconscious biases and the influence of these on clinical practice could be especially pertinent for improving care within commonly stigmatised conditions, such as obesity.

## supplementary material

10.1136/bmjopen-2024-086722 online supplemental file 1

## Data Availability

All data relevant to the study are included in the article or uploaded as supplementary information.
