# Supplementary material for: ‘My first thoughts are…’: a Framework Method analysis of UK general practice healthcare professionals’ internal dialogue and clinical reasoning processes when seeing patients living with obesity in primary care
Source: BMJ Open. 2025 Apr 2;15(4):e086722. doi: 10.1136/bmjopen-2024-086722 (PMC11966948; doi:10.1136/bmjopen-2024-086722)
Supplement: Supplementary file 2 [file bmjopen-2024-086722.reviewer_comments.pdf]

PEER REVIEW HISTORY

BMJ Open publishes all reviews undertaken for accepted manuscripts. Reviewers are asked to complete a checklist review form and are provided with free text boxes to elaborate on their assessment. These free text comments are reproduced below.

ARTICLE DETAILS

Title (Provisional)

'My first thoughts are...': a Framework Method analysis of general practice healthcare professionals' internal dialogue and clinical reasoning processes when seeing patients living with obesity in primary care

Authors

Serjeant, Sarah; Abbott, Sally; Parretti, Helen; Greenfield, Sheila

---

VERSION 1 - REVIEW

**Reviewer**	**1**
**Name**	**Kraftson, Andrew T**
**Affiliation**	**University of Michigan, Division of Metabolism, Endocrinology and Diabetes**
**Date**	**17-Jun-2024**
**COI**	**none**

This is a very well-written paper. As an endocrinologist who specializes in obesity medicine, the descriptive comments are important to illuminate (albeit difficult to read due to the comments being rife with stereotypical thinking). This is important work that would be valuable for practitioners and trainees to view.

However, I do hope there is a follow-up to this study. There is the implication that the comments/attitudes will cause clinical inertia and may influence diagnostic/therapeutic recommendations. However, the veracity of this could/should be examined. For example: participant is given the patient-case example, shown photo, asked questions/impressions, and then provided with diagnostic/therapeutic choices that would reflect standards-of-care practices (eg: are certain labs/tests obtained, are certain therapeutic options discussed, are appropriate referrals placed, etc). I think this is an important aspect that is missing from the current study.

However, I know this was not the "goal", per se, of the paper - but the overall impact of the work, as presented, is diminished without the exploration of the true clinical implications of these attitudes. We should not assume that the level of care received based on attitudes, alone.

**Reviewer**	**2**
**Name**	**Holtrop, Jodi Summers**
**Affiliation**	**University of Colorado Anschutz Medical Campus**
**Date**	**07-Oct-2024**
**COI**	**None**

This study was done quite some time ago (2019). Not a fatal flaw but opinions might have changed since then.

It would be better if a brief description of the sampling strategy was included rather than referring to another paper.

The Framework Method of analysis needs to be described. Is it the same thing as Matrix Analysis, just by a different name? A reference or two to this method is also needed.

Spell out VoIP. I'm guessing this is the video conferencing software?

When reading the results of the themes I was wondering about the interviews and how they were set up. This section is very short and more detail could be added such as what actual questions were asked. The vignettes show what they were looking at but how were the participants prompted? Were the participants prodded to come up with what they thought might be happening or just their first thoughts as they looked at the pictures or something else?

The results are interesting and the quotes help illustrate the main points.

Literature on stigma should be included and is not.

Need to include limitations. This was conducted with only a few HCPs from the UK so the generalizability of the results is very limited. However it does point to areas to look for consideration of stigma. Also using pictures and not real people is an obvious limitation and should be noted.

Overall appreciate the authors brevity, but more detail is needed to understand what exactly was done.

VERSION 1 - AUTHOR RESPONSE

Here is a point-by-point response to Reviewer 1's comments:

Comment 1: This is a very well-written paper. As an endocrinologist who specializes in obesity medicine, the descriptive comments are important to illuminate (albeit difficult to read due to the comments being rife with stereotypical thinking). This is important work that would be valuable for practitioners and trainees to view. However, I do hope there is a follow-up to this study. There is the implication that the comments/attitudes will cause clinical inertia and may influence diagnostic/therapeutic recommendations. However, the veracity of this could/should

be examined. For example: participant is given the patient-case example, shown photo, asked questions/impressions, and then provided with diagnostic/therapeutic choices that would reflect standards-of-care practices (eg: are certain labs/tests obtained, are certain therapeutic options discussed, are appropriate referrals placed, etc). I think this is an important aspect that is missing from the current study. However, I know this was not the "goal", per se, of the paper - but the overall impact of the work, as presented, is diminished without the exploration of the true clinical implications of these attitudes. We should not assume that the level of care received based on attitudes, alone.

Response 1: We would like to thank Reviewer 1 for his positive comments about the paper and we are pleased that he considers it to be important work. Our interviews included both first impressions and treatment considerations, however due to the volume of data produced, the current paper focuses on the former to allow depth of discussion. We hope to build on this work in the future, which may include a follow-up paper focusing on our data regarding treatment decisions. In the meantime, statements have been added to p.10 & 11 around the need for future research on this topic.

Here is a point-by-point response to Reviewer 2's comments:

Comment 1: This study was done quite some time ago (2019). Not a fatal flaw but opinions might have changed since then.

Response 1: Thank you, we have acknowledged the age of the data as described above in our responses to the Editor's comments (see p.2 & 11).

Comment 2: It would be better if a brief description of the sampling strategy was included rather than referring to another paper.

Response 2: Thank you, we have added this description as described above in our responses to the Editor's comments (see p.3, sampling strategy sub-section).

Comment 3: The Framework Method of analysis needs to be described. Is it the same thing as Matrix Analysis, just by a different name? A reference or two to this method is also needed.

Response 3: Thank you, further description has been added on the Framework Method on p.3 (data analysis sub-section). Reference #17 is the citation for this analytical method.

Comment 4: Spell out VoIP. I'm guessing this is the video conferencing software?

Response 4: Apologies this was not clear, this has now been further described on p.3 (data collection sub-section).

Comment 5: When reading the results of the themes I was wondering about the interviews and how they were set up. This section is very short and more detail could be added such as what actual questions were asked. The vignettes show what they were looking at but how were the participants prompted? Were the participants prodded to come up with what they thought might be happening or just their first thoughts as they looked at the pictures or something else?

Response 5: The interview guide that accompanied the vignettes has now been added to the supplemental material and is cited in the main body alongside the vignettes (p.3, data collection sub-section).
Update 26/02 – The Editorial Office asked us to remove the vignette images from the submission

due to copyright. This has been actioned, and the vignettes are no longer provided within the supplementary information, however the interview guide is there.

Comment 6: The results are interesting and the quotes help illustrate the main points.

Response 6: Thank you, we are pleased that the reviewer found the results interesting and quotes illustrative.

Comment 7: Literature on stigma should be included and is not.

Response 7: Thank you for highlighting this. Literature on stigma in primary care has been further explored and an addition has been made within the discussion section, p.9 & 10.

Comment 8: Need to include limitations. This was conducted with only a few HCPs from the UK so the generalizability of the results is very limited. However it does point to areas to look for consideration of stigma.

Response 8: We have critiqued limitations further, acknowledging that findings may not be transferrable outside the UK (p.10-11). Our sample size of HCPs was determined based on the principle of data saturation. Data saturation was reached within the sample of 20 participants, whereby additional data collection yielded no new themes or insights.

Comment 9: Also using pictures and not real people is an obvious limitation and should be noted.

Response 9: We have strengthened our comment acknowledging this as a limitation within the manuscript on p.1 & 11.

Comment 10: Overall appreciate the authors brevity, but more detail is needed to understand what exactly was done.

Response 10: Thank you, we hope in the above edits and changes to the manuscript have now addressed this comment.
